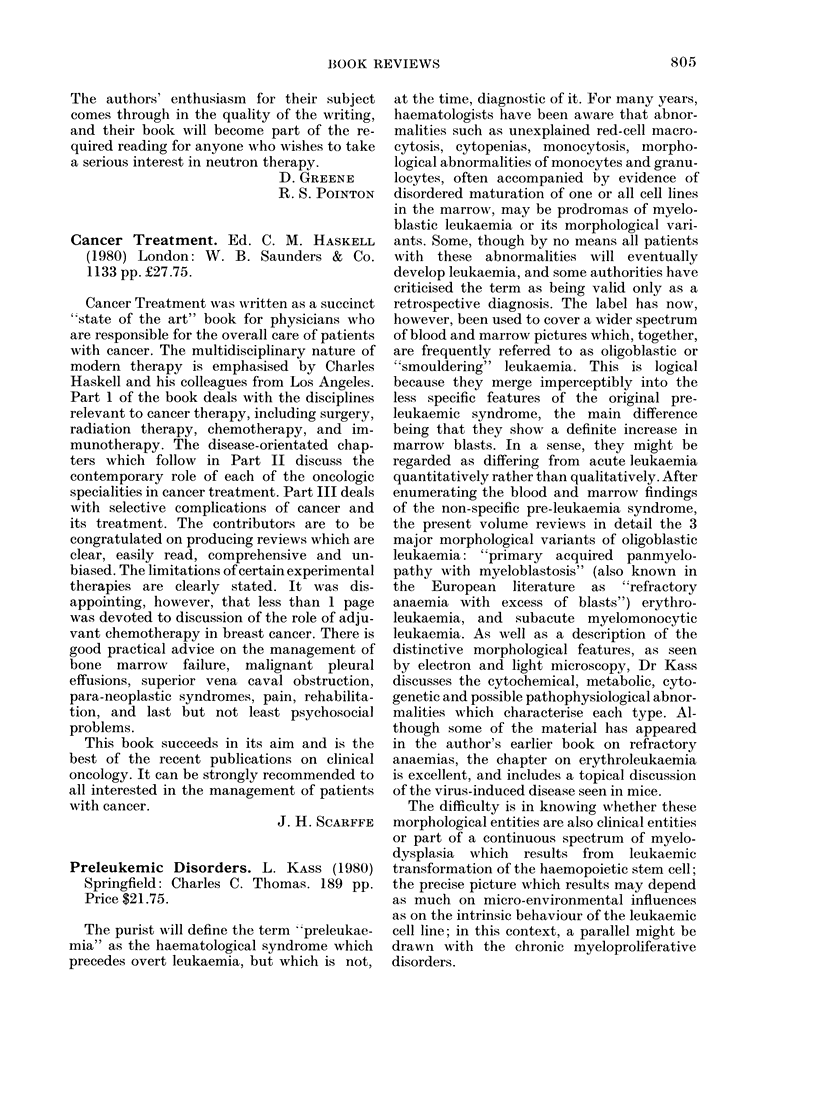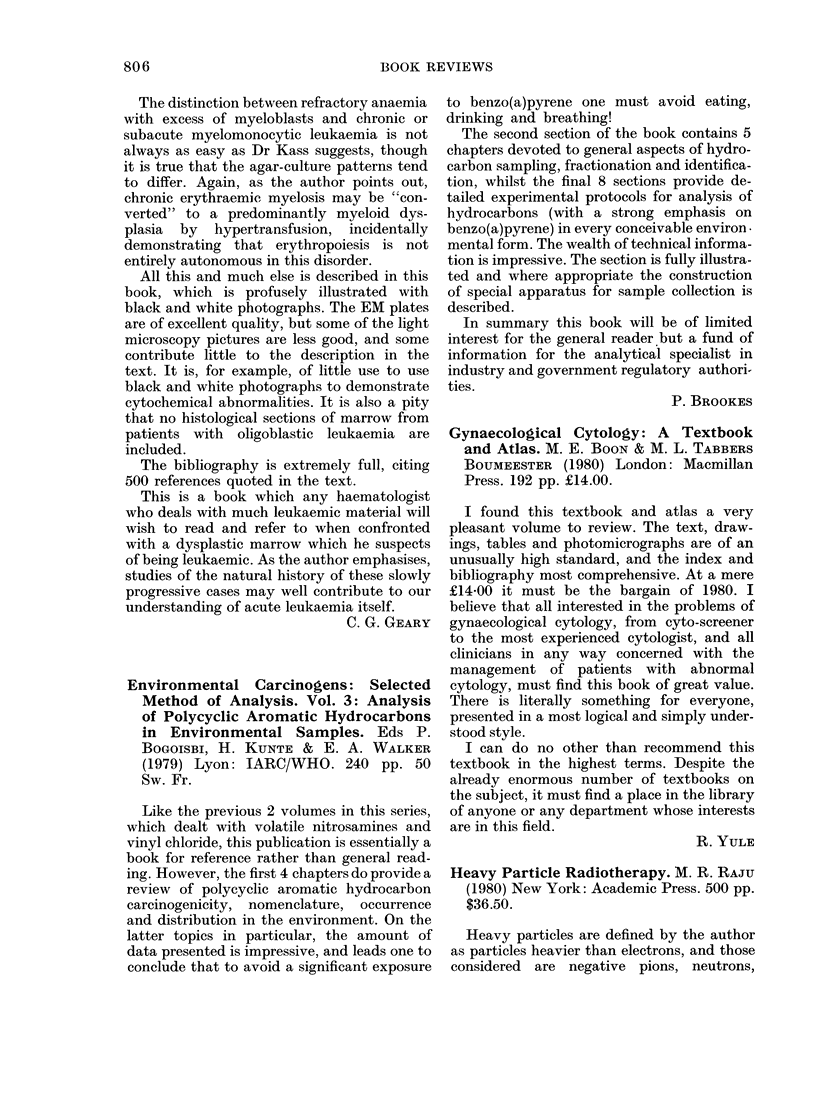# Preleukemic Disorders

**Published:** 1980-11

**Authors:** C. G. Geary


**Preleukemic Disorders.** L. KASS (1980) Springfield: Charles C. Thomas. 189 pp. Price $21.75.

The purist will define the term "preleukaemia" as the haematological syndrome which precedes overt leukaemia, but which is not, at the time, diagnostic of it. For many years, haematologists have been aware that abnormalities such as unexplained red-cell macrocytosis, cytopenias, monocytosis, morphological abnormalities of monocytes and granulocytes, often accompanied by evidence of disordered maturation of one or all cell lines in the marrow, may be prodromas of myeloblastic leukaemia or its morphological variants. Some, though by no means all patients with these abnormalities will eventually develop leukaemia, and some authorities have criticised the term as being valid only as a retrospective diagnosis. The label has now, however, been used to cover a wider spectrum of blood and marrow pictures which, together, are frequently referred to as oligoblastic or "smouldering" leukaemia. This is logical because they merge imperceptibly into the less specific features of the original preleukaemic syndrome, the main difference being that they show a definite increase in marrow blasts. In a sense, they might be regarded as differing from acute leukaemia quantitatively rather than qualitatively. After enumerating the blood and marrow findings of the non-specific pre-leukaemia syndrome, the present volume reviews in detail the 3 major morphological variants of oligoblastic leukaemia: "primary acquired panmyelopathy with myeloblastosis" (also known in the European literature as "refractory anaemia with excess of blasts") erythroleukaemia, and subacute myelomonocytic leukaemia. As well as a description of the distinctive morphological features, as seen by electron and light microscopy, Dr Kass discusses the cytochemical, metabolic, cytogenetic and possible pathophysiological abnormalities which characterise each type. Although some of the material has appeared in the author's earlier book on refractory anaemias, the chapter on erythroleukaemia is excellent, and includes a topical discussion of the virus-induced disease seen in mice.

The difficulty is in knowing whether these morphological entities are also clinical entities or part of a continuous spectrum of myelodysplasia which results from leukaemic transformation of the haemopoietic stem cell; the precise picture which results may depend as much on micro-environmental influences as on the intrinsic behaviour of the leukaemic cell line; in this context, a parallel might be drawn with the chronic myeloproliferative disorders.

The distinction between refractory anaemia with excess of myeloblasts and chronic or subacute myelomonocytic leukaemia is not always as easy as Dr Kass suggests, though it is true that the agar-culture patterns tend to differ. Again, as the author points out, chronic erythraemic myelosis may be "converted" to a predominantly myeloid dysplasia by hypertransfusion, incidentally demonstrating that erythropoiesis is not entirely autonomous in this disorder.

All this and much else is described in this book, which is profusely illustrated with black and white photographs. The EM plates are of excellent quality, but some of the light microscopy pictures are less good, and some contribute little to the description in the text. It is, for example, of little use to use black and white photographs to demonstrate cytochemical abnormalities. It is also a pity that no histological sections of marrow from patients with oligoblastic leukaemia are included.

The bibliography is extremely full, citing 500 references quoted in the text.

This is a book which any haematologist who deals with much leukaemic material will wish to read and refer to when confronted with a dysplastic marrow which he suspects of being leukaemic. As the author emphasises, studies of the natural history of these slowly progressive cases may well contribute to our understanding of acute leukaemia itself.

C. G. Geary